# *Caenorhabditis elegans* as a Model to Study Aging and Photoaging

**DOI:** 10.3390/biom14101235

**Published:** 2024-09-30

**Authors:** Saowanee Jeayeng, Jirapan Thongsroy, Sirithip Chuaijit

**Affiliations:** 1 Department of Medical Sciences, School of Medicine, Walailak University, Nakhon Si Thammarat 80161, Thailand; saowanee.j10@gmail.com (S.J.); jirapan.th@wu.ac.th (J.T.); 2 Research Center in Tropical Pathobiology, Walailak University, Nakhon Si Thammarat 80161, Thailand

**Keywords:** *Caenorhabditis elegans*, photoaging, chronological aging, UV radiation, longevity

## Abstract

*Caenorhabditis elegans* (*C. elegans*) has emerged as an outstanding model organism for investigating the aging process due to its shortened lifespan, well-defined genome, and accessibility of potent genetic tools. This review presents the current findings on chronological aging and photoaging in *C. elegans*, exploring the elaborate molecular pathways that control these processes. The progression of chronological aging is characterized by a gradual deterioration of physiological functions and is influenced by an interaction of genetic and environmental factors, including the insulin/insulin-like signaling (IIS) pathway. In contrast, photoaging is characterized by increased oxidative stress, DNA damage, and activation of stress response pathways induced by UV exposure. Although the genetic mechanisms of chronological aging in *C. elegans* have been characterized by extensive research, the pathways regulating photoaging are comparatively less well-studied. Here, we provide an overview of the current understanding of aging research, including the crucial genes and genetic pathways involved in the aging and photoaging processes of *C. elegans*. Understanding the complex interactions between these factors will provide invaluable insights into the molecular mechanisms underlying chronological aging and photoaging and may lead to novel therapeutic approaches and further studies for promoting healthy aging in humans.

## 1. Introduction

*Caenorhabditis elegans* (*C. elegans*) has emerged as a useful model organism for the study of aging due to its numerous advantages. Its short lifespan, well-characterized genome, and the availability of advanced genetic tools make this nematode an exceptionally valuable system for investigating the complex mechanisms underlying both chronological and photoaging processes. This review provides a comprehensive examination of the current understanding of these two fundamental aspects of aging in *C. elegans* and explores the elaborate molecular pathways that underlie these mechanisms.

Chronological aging in *C. elegans* is characterized by a progressive deterioration in physiological functions and the occurrence of various age-related diseases. These include the deterioration of important tissues and organ systems, such as the pharynx, intestine, and nervous system. This gradual deterioration of organismal integrity is influenced by a complex interplay of genetic and environmental factors. One of the most extensively studied signaling pathways regulating longevity in *C. elegans* is the insulin/insulin-like signaling (IIS) pathway [1]. Mutations in DAF-2, which reduce insulin/IGF-1 signaling, generally extend lifespan by activating DAF-16, a key transcription factor involved in stress resistance and longevity pathways. This indicates the central role of the IIS pathway in the regulation of aging and longevity processes [2,3]. In addition, the mechanistic Target of Rapamycin (mTOR) pathway regulates processes such as protein synthesis and autophagy through the integration of nutrient and growth signals, and its inhibition can extend lifespan by enhancing autophagy and improving mitochondrial function [4]. Additionally, other genetic and environmental determinants, such as dietary intake, temperature, and exposure to various stressors, have also been found to influence the rate of chronological aging in *C. elegans* [5]. The various factors involved highlight the complexity of the aging process and emphasize the importance of *C. elegans* as a model for understanding the molecular mechanisms that control it.

Photoaging is the process of premature skin aging caused by repeated exposure to ultraviolet (UV) radiation, mainly from the sun. This phenomenon, characterized by wrinkles, fine lines, age spots, and a loss of skin elasticity, has been studied extensively in humans. Photoaging is characterized by increased oxidative stress, DNA damage, and the activation of various stress response pathways, all of which contribute to premature cellular senescence [6,7]. Despite the comprehensive characterization of the genetic mechanisms regulating chronological aging in *C. elegans*, the pathways controlling photoaging remain relatively less understood. However, recent evidence suggests that mitochondrial function and specific stress response mechanisms play an important role in modulating the photoaging process. In addition, activation of conserved stress response pathways, such as activation of heat shock proteins and the DAF-16/FOXO transcription factor, has been shown to influence an organism’s susceptibility to photoaging [8]. Moreover, the Mitogen-Activated Protein Kinase (MAPK) pathway has also been investigated [9]. This review has shed light on the complex interplay between genetic factors, environmental stressors, and the basic biology of the aging process, providing the basis for a more comprehensive understanding of how organisms cope with the biological effects of chronological aging and photoaging. The results of the *C. elegans* studies have the potential to inform and stimulate future investigations into the molecular basis of aging in more complex organisms, ultimately contributing to the development of strategies to promote healthy longevity.

## 2. Chronological Aging in *Caenorhabditis elegans*

*C. elegans* has proven to be a powerful model in the field of aging research due to its numerous advantages that facilitate the study of the complex aging process. The short lifespan of *C. elegans*, typically around three weeks, allows researchers to efficiently observe and analyze the complete aging pathway within a manageable period. The nematode’s concise and fully sequenced genome, in combination with well-established genetic tools, enables precise manipulation and investigation of genes and pathways associated with aging [10,11]. The transparency of *C. elegans* further enhances its utility by allowing real-time visualization of cellular and molecular events throughout its lifespan. Additionally, the ease of culturing and maintaining large populations of *C. elegans* at a low maintenance cost makes it an accessible and efficient model for high-throughput screening of potential anti-aging compounds. The combination of these characteristics has established *C. elegans* as a foundation for aging research, enabling scientists to gain valuable insights into the genetic, molecular, and environmental factors that influence lifespan and healthspan [12,13,14]. Numerous studies have shown that genetic, environmental, and pharmacological interventions can significantly influence the aging process in *C. elegans*. Overall, the study of aging in *C. elegans* has provided significant insights into the molecular, genetic, and environmental factors that influence aging, with many findings being relevant to understanding human aging and developing interventions to promote health.

### 2.1. Molecular Mechanism Insights into Chronological Aging in C. elegans

Aging of *C. elegans* is a complex process influenced by various molecular mechanisms that interact across different biological scales. One of the primary hallmarks of aging in *C. elegans* is the diminishment in cellular function. The IIS pathway, in particular the insulin receptor ortholog DAF-2, has been studied [15]. In this signaling pathway, the DAF-2 receptor, upon binding to insulin-like ligands, initiates a cascade that results in the phosphorylation and cytoplasmic retention of the transcription factor DAF-16, a homolog of the mammalian Forkhead box O transcription factor (FOXO), and is a key regulator of stress resistance and longevity [16]. Mutations that reduce IIS activity, such as in daf-2 or age-1, allow DAF-16 to translocate to the nucleus, where it activates genes promoting stress resistance, detoxification, and longevity [2]. Furthermore, host Cell Factor-1 (HCF-1), a nuclear co-repressor of DAF-16, has been demonstrated to function downstream of the sirtuin 1 (SIRT1) ortholog SIR-2.1 in *C. elegans* to modulate lifespan. HCF-1 physically interacts with DAF-16, influencing the expression of DAF-16-regulated genes, thereby impacting lifespan [17]. In addition, c-Jun N-terminal Kinase pathway (JNK-1), another member of the MAPK family, directly phosphorylates DAF-16, promoting its nuclear translocation and the activation of stress-resistant genes. This phosphorylation by JNK-1 represents a crucial point where multiple signaling pathways, including IIS and SIR-2.1, converge to regulate DAF-16 activity. While IIS negatively regulates DAF-16, both SIR-2.1 and JNK-1 positively influence its activation, thereby promoting longevity and stress resistance [18].

Epigenetic mechanisms, such as histone modifications, also play a significant role in the aging process. For example, the demethylase UTX-1 influences lifespan by targeting genes in the IIS pathway and affecting DAF-16 levels [19]. Dietary restriction (DR) has been shown to extend the lifespan of *C. elegans*, potentially by reducing IIS and metabolic rate. Furthermore, SIR-2.1 requires NAD+ as a cofactor, with NAD+ levels being closely associated with mitochondrial health and function, which are modulated by calorie restriction. The study also reveals that NAD+ depletion in DNA repair-deficient *C. elegans* models causes mitochondrial dysfunction and accelerated aging, highlighting a potential link between DNA repair, NAD+ levels, and the SIRT-2.1 pathway in the context of calorie restriction [20,21,22].

AMP-activated protein kinase (AMPK), encoded by the *aak-2* gene in *C. elegans*, acts as an energy sensor, promoting ATP production when energy levels are low. AMPK activation can extend lifespan by phosphorylating and activating DAF-16, as well as by influencing mitochondrial function and autophagy [23,24]. The mTOR pathway is another important regulator, integrating nutrient and growth factor signals to control processes such as protein synthesis and autophagy. Inhibition of mTOR can increase lifespan, possibly by enhancing autophagy and modulating mitochondrial function [4,25].

Autophagy, a crucial cellular process for maintaining homeostasis and promoting longevity, is tightly regulated by various transcription factors across species. Among these, HLH-30, the *C. elegans* ortholog of the mammalian transcription factor EB (TFEB), plays a crucial role. HLH-30/TFEB belongs to the basic helix-loop-helix leucine-zipper (bHLH-Zip) transcription factor family, which is characterized by its ability to bind DNA and regulate gene expression, which is important for autophagic processes [26]. In a nutrient-rich environment in *C. elgans*, LET-363/mTOR is anchored to the lysosomal membrane and activated by the protein Rheb (Ras homolog enriched in the brain). Rheb’s activity is regulated by the tuberous sclerosis (TSC) protein complex, which responds to signals reflecting the cell’s metabolic status. Activation of LET-363/mTOR results in the phosphorylation and inactivation of transcription factors such as DAF-16/FOXO and HLH-30/TFEB, thereby preventing their translocation into the nucleus. Conversely, when nutrient levels are low, the TSC complex inhibits Rheb, leading to the inactivation of LET-363/mTOR, which then detaches from the lysosomal membrane. In the deficiency of LET-363/mTOR phosphorylation, DAF-16/FOXO and HLH-30/TFEB can enter the nucleus and activate the transcription of genes, including those encoding proteins essential for autophagy [27]. Proteostasis, or protein homeostasis, also deteriorates with age, leading to the accumulation of protein aggregates in specific tissues. This is regulated by core protein quality control systems such as chaperones, the proteasome, and autophagy, which exhibit tissue-specific effects on protein aggregation. For instance, myosin function, facilitated by its chaperones UNC-45 and HSP-90, is crucial in maintaining muscle integrity and preventing sarcopenia. Age-related reduction and degradation of these chaperone proteins are key contributors to muscle aging [28].

**Figure 1:**
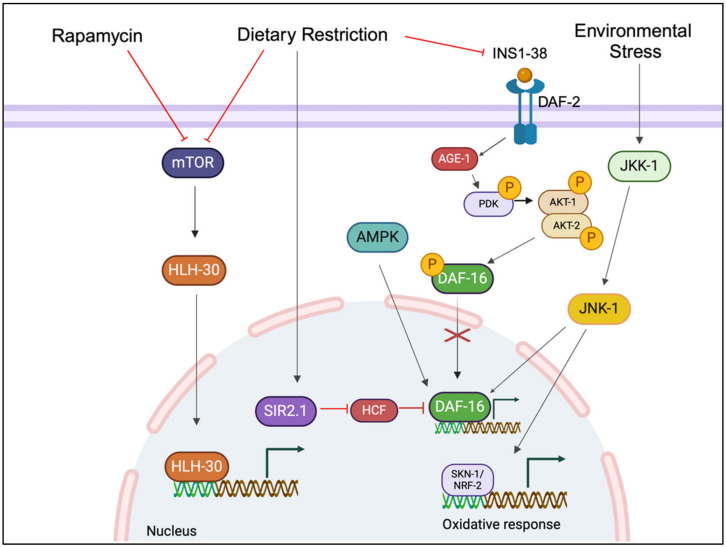
The figure illustrates the integrated pathways that regulate aging in *C. elegans*, with DAF-16/FOXO as the central transcription factor promoting longevity. The insulin/IGF signaling (IIS) pathway, when active, prevents DAF-16/FOXO from entering the nucleus, thereby inhibiting its function. However, blocking the IIS pathway allows DAF-16/FOXO to translocate to the nucleus, where it activates genes that extend lifespan. Surrounding pathways, such as AMPK and JNK, are shown to activate DAF-16/FOXO. Additionally, SKN-1 is regulated by the p38 mitogen-activated protein kinase (MAPK) pathway through JKK-1 and JNK-1. Moreover, the SIRT1 pathway activates DAF-16/FOXO. Dietary restriction (DR) inhibits both the IIS and mTOR signaling pathways, suppression of this process leads to increased autophagy and a reduction in translation. By inhibiting mTOR, HLH-30/TFEB can enter the nucleus and activate the transcription of genes, including those encoding proteins necessary for autophagy.

The transcription factor PHA-4/FOXA plays an important role in mediating longevity under conditions where nutrient-sensing pathways, particularly LET-363/mTOR, are downregulated. Under dietary restriction (DR), PHA-4/FOXA translocates to the nucleus, where it drives the expression of genes essential for oxidative stress resistance, including the superoxide dismutase genes *sod-1*, *sod-2*, *sod-4*, and *sod-5*. These genes play an important role in mitigating the damaging effects of reactive oxygen species (ROS), thereby protecting cellular integrity during starvation [29]. Moreover, PHA-4/FOXA is crucial for the induction of autophagy, a cellular recycling process that is essential for maintaining homeostasis under DR conditions [30]. This is particularly evident in eat-2 mutants, a genetic model of DR in *C. elegans*, where PHA-4/FOXA activity is necessary for the longevity phenotype. The ability of PHA-4/FOXA to coordinate oxidative stress resistance and autophagy highlights its critical role in modulating lifespan in response to food intake. In *C. elegans*, the transcription factor SKN-1, which corresponds to the mammalian Nuclear factor erythroid 2-related factor (Nrf2) protein, plays a crucial role in regulating the oxidative stress response. SKN-1 is crucial for maintaining protein homeostasis and promoting longevity. Additionally, SKN-1 is regulated by the p38 MAPK pathway. SKN-1/Nrf2 also plays an important role in dietary restriction (DR)-mediated longevity. This factor is closely linked to the IIS pathway. Under conditions of reduced IIS, SKN-1/Nrf2 accumulates in the nucleus of intestinal cells, where it activates genes involved in the phase II detoxification system. This system is essential for neutralizing free oxygen radicals and other harmful byproducts of metabolism, thereby conferring resistance to oxidative stress [31,32,33,34]. The important signaling pathways and genes that regulate aging are shown in Figure 1 and Table 1.

Understanding these mechanisms in *C. elegans* provides valuable insights into the aging process and may pave the way for interventions to promote healthier aging in humans.

### 2.2. Impaired DNA Repair Mechanisms and Genomic Instability during Aging in Caenorhabditis elegans

Impaired DNA repair mechanisms are a significant hallmark of aging in *C. elegans*, leading to a gradual decrease in the efficiency of genome maintenance, which is often attributed to defects in DNA repair mechanisms. The spectrum of phenotypic consequences of this instability is broad and includes severe developmental defects and more subtle disorders [45]. The concept of ‘segmental progeria’, which describes DNA repair diseases that exhibit some but not all features of premature aging, emphasizes the contribution of inefficient DNA repair to both aging and neurodegeneration [46,47]. It is also important to note that genomic instability can act as a catalyst, exacerbating or even driving other hallmarks of aging, such as telomere degradation and mitochondrial dysfunction [48].

The nematode *C. elegans* has proven to be a valuable model for studying DNA repair as the major DNA repair pathways are conserved between *C. elegans* and higher organisms [49]. Most of our current understanding of DNA repair in *C. elegans* comes from studies of germline genome stability, as the germline represents the only tissue in adult worms where cells continue to divide [50]. The phenotypic endpoints of DNA repair in the germline, such as survival rates, brood size, and male abundance (a marker for meiotic crossover defects), can be readily monitored [51]. Additionally, immunohistochemistry and fluorescence-based reporters provide standardized tools for observing apoptosis and the activation of DNA damage response signaling in germline cells and embryos [50]. Direct measurements of nuclear and mitochondrial DNA damage from whole worm extracts are also possible, although germline effects may predominate due to the higher number of germline nuclei compared to somatic cells [52,53]. The comet assay serves as another tool for measuring DNA single- or double-strand breaks in germline nuclei or embryos [54].

In the context of post-mitotic neurons, that do not undergo DNA replication, repair pathways that are not directly coupled to replication, such as base excision repair (BER), nucleotide excision repair (NER), and non-homologous end joining (NHEJ), are particularly important for the maintenance of genomic integrity [55]. NER, a versatile pathway for repairing helix-distorting DNA adducts, has been extensively studied in *C. elegans*, with both the transcription-coupled (TC-NER) and global genome NER (GG-NER) pathways being active [56]. The BER pathway, responsible for repairing non-helix-distorting chemically modified DNA bases, is less well explored in *C. elegans* [57]. Key factors in this pathway include DNA glycosylases (UNG-1 and NTH-1), AP endonucleases (EXO-3 and APN-1), and DNA polymerase θ [57,58,59,60,61,62]. The repair of highly deleterious double-strand breaks (DSBs) in *C. elegans* is mainly accomplished by error-free homologous recombination (HR) or error-prone non-homologous end joining (NHEJ) or single-strand annealing (SSA) in *C. elegans*, whereas DNA replication errors are corrected by mismatch repair (MMR) [63,64]. In addition, the accumulation of defective mtDNA increases with maternal age, and some of this defective mtDNA is passed on to offspring, indicating an age-dependent mechanism of mtDNA quality control influenced by regulators of programmed cell death (PCD) and aging [65]. The mitochondrial free radical theory of aging (mFRTA) states that oxidative damage to mtDNA accumulates with age, although the relationship between mtDNA damage and lifespan is complex and not directly proportional [66]. Moreover, a transcriptomic analysis of aging in *C. elegans* revealed an age-related downregulation of genes involved in DNA repair pathways and an increase in misprocessed RNAs that may contribute to both genomic instability and mitochondrial dysfunction [67]. Overall, these findings highlight the multifaceted nature of DNA repair impairments in aging *C. elegans*, affecting both the nuclear and mitochondrial genomes, and highlight the broader implications for organismal aging and healthspan.

## 3. Photoaging in *Caenorhabditis elegans*

Ultraviolet (UV) radiation, a part of sunlight, is divided into three types: UVA, UV-B, and UVC. UVA (320–400 nm) penetrates deeply into the skin, causing oxidative stress and contributing to photoaging and skin cancer. UVB (290–320 nm) has higher energy, leading to direct DNA damage, sunburn, and plays an important role in non-melanoma skin cancers. UVC (100–290 nm), the most harmful, is absorbed by the ozone layer but can cause severe DNA damage when emitted from artificial sources [68,69,70]. Understanding the distinct effects of each UV type is essential for protecting against its harmful effects.

**Figure 2:**
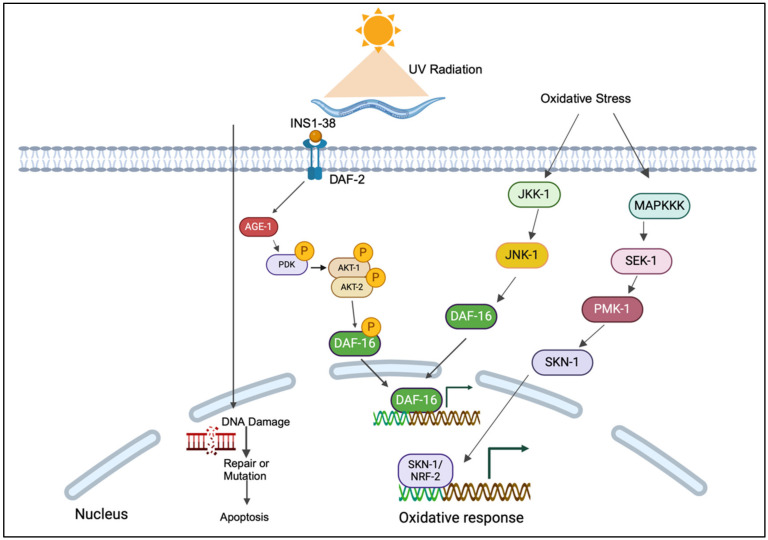
The figure illustrates the complex molecular pathways that *C. elegans* employs to respond to UV radiation and oxidative stress. The focus is on the DAF-2/DAF-16 insulin/IGF-1 signaling pathway. Upon exposure to UV radiation, which induces DNA damage, the insulin-like peptide INS-1-38 activates the DAF-2 receptor and triggers a cascade involving AGE-1, PDK-1, and AKT-1/2. This leads to phosphorylation and cytoplasmic retention of the DAF-16 transcription factor. However, under conditions of reduced insulin signaling or increased stress, DAF-16 translocates to the nucleus, where it promotes the expression of genes involved in DNA repair, stress resistance, and longevity. Concurrently, oxidative stress activates the signaling pathways JNK-1 and PMK-1, further enhancing DAF-16 activity and leading to a robust response to oxidative stress. The transcription factor SKN-1, a homolog of NRF-2 in mammals, also plays a crucial role by collaborating with DAF-16 to regulate antioxidant gene expression, thereby protecting the organism from oxidative damage caused by UV radiation.

Photoaging, the premature aging of biological tissues by UV radiation, is an important area of research to understand the mechanisms of aging. *C. elegans* is an outstanding model organism for studying the molecular and cellular effects of UV radiation because its genetics are well-mapped, it is transparent, and several biological signaling pathways relevant to aging are conserved. Exposure to UVA is an important cause of photoaging, and studies have been carried out using the model organism *C. elegans.* Previous studies using the model organism *C. elegans* have elucidated various molecular mechanisms. For example, Prasanth et al. indicated that UVA induces premature aging characterized by increased reactive oxygen species (ROS) and collagen damage, primarily mediated through the p38 and JNK pathways of the MAPK signaling cascade [71]. Additionally, UVA exposure accelerates aging via an insulin-like signaling pathway, affecting behaviors such as pharyngeal movements and brood size, while also damaging worms’ neuronal network [8]. UVB radiation causes photoaging in *C. elegans* primarily through the formation of ROS and direct DNA damage such as cyclobutane pyrimidine dimers (CPDs) and 6-4 photoproducts. These lesions impair DNA replication and transcription and trigger a cascade of cellular stress responses. This type of damage is primarily repaired by the NER pathway [72]. Deficiencies of NER components, such as XPA-1 and CSB-1, significantly increase sensitivity to UVB radiation, leading to accelerated aging characterized by developmental delays, shortened lifespan, and increased apoptotic activity, particularly in the germline. UVB-induced damage also activates the IIS pathway, with DAF-16/FOXO playing a protective role by enhancing stress response and longevity genes. However, prolonged UVB exposure can disrupt this pathway, further contributing to the aging process. Physiological deterioration, such as impaired locomotion and reduced reproductive performance, emphasizes the aging effects of UVB in *C. elegans* [56,73]. Several antioxidants have been shown to rescue *C. elegans* from UV-specific damage, protecting against photoaging and UV-induced stress. Lin et al. revealed that astaxanthin (AST), a xanthophyll carotenoid, mitigated UV-induced aging by improving survival rates, reducing aging biomarkers, and alleviating mitochondrial dysfunction through the activation of the JNK-1/DAF-16 pathway [74]. Under UVA exposure, *C. elegans* treated with xanthotoxin showed improvements in various aspects, including hatchability, body size, and brood size, as well as restoration of behaviors such as head thrashing and body bending. Oxidative stress was reduced, and the upregulation of apoptosis-related genes (*ced-3* and *ced-4*) was mitigated [75]. Prasanth et al. investigated the synergistic potential of antioxidants, including green tea extract, naringenin, and naringin, in mitigating the adverse effects of photoaging [76]. Didymin, a flavonoid compound, has been shown to protect *C. elegans* from UV-induced stress by reducing ROS levels and increasing superoxide dismutase (SOD) activity. However, certain mutant strains, including *daf-16*, *akt-1*, *akt-2*, and *age-1*, remain susceptible to UV irradiation even after didymin treatment. In contrast, the *daf-2(e1371)* mutant showed resistance, suggesting a complex interaction between didymin and the insulin-like signaling pathway in UV stress resistance [77]. Overall, these studies emphasized the complexity of the interactions between UV-induced damage, antioxidant response, and the key pathways that regulate stress resistance and aging in *C. elegans*. The critical pathways and genes that regulate photoaging are illustrated in Figure 2.

### 3.1. Molecular Mechanism of UV-Induced Aging in Caenorhabditis elegans

UV radiation is a significant factor in inducing aging, particularly through mechanisms involving DNA damage and oxidative stress, as observed in the model organism *C. elegans*. The molecular mechanisms underlying UV-induced aging in *C. elegans* are complex and involve several important signaling pathways and cellular responses. One important signaling pathway is the MAPK pathway, which includes both p38 and JNK MAPKs. UV radiation activates the PMK-1/p38 MAPK signaling pathway via the upstream regulators JKK-1 and MOM-4, leading to phosphorylation and activation of PMK-1, which is crucial for the organism’s response to UV stress [9]. Additionally, the JNK-MAPK pathway is also involved, as shown by the reduced survival of *jnk-1* mutants under UV exposure, highlighting its role in mediating the anti-aging effects through the JNK-1/DAF-16 signaling pathway [74]. The DAF-16 transcription factor, a key factor in the IIS pathway, is also critical in this context. UVA exposure accelerates aging by affecting collagen synthesis and function, which is monitored using *col-19*: GFP strains. This process depends on the insulin-like signaling pathway, as shown by the altered behavior and reduced lifespan in *daf-2* mutants [78]. Moreover, non-coding RNAs (ncRNAs), including miRNAs, are differentially regulated in response to UV irradiation, influencing MAPK signaling, NER, and proteasome activity, thereby contributing to the cellular response to UV damage [79]. Overall, these studies emphasize the complexity of the molecular mechanisms underlying UV-induced aging in *C. elegans*, involving a network of signaling pathways, DNA repair mechanisms, and regulatory RNAs that work together to cope with and mitigate the deleterious effects of UV radiation.

### 3.2. Impaired DNA Repair in Photoaging and UV-Induced Damage in Caenorhabditis elegans

UV radiation is a potent genotoxic agent that significantly impairs DNA integrity in *C. elegans*. NER-deficient larval worms showed increased sensitivity to UV radiation, highlighting the critical role of NER in protecting against UV-induced damage. Within the NER pathway, transcription-coupled NER (TC-NER) was found to be particularly crucial for survival and growth following UV exposure, whereas global genomic NER (GG-NER) played a lesser role unless TC-NER was deficient. This distinction suggests that TC-NER provides a more direct and targeted response to UV-induced lesions, particularly in genes that are actively transcribed, while GG-NER serves as a secondary line of defense when TC-NER is impaired [56]. UV-induced DNA damage in *C. elegans* also triggers a complex network of molecular responses, including cell cycle arrest, DNA repair, and apoptosis [80]. Specifically, UVC-induced DNA lesions are processed by the NER pathway, which subsequently activates the homologous recombination (HR) pathway and downstream checkpoint kinases, leading to apoptosis in meiotic germ cells [81]. UVB radiation, a component of solar UV, is particularly damaging as it causes significant DNA damage and requires robust repair mechanisms such as photoreactivation and excision repair [9]. Far-UVC (222 nm) and conventional UVC (254 nm) have different biological effects on *C. elegans*. While 222-UVC causes severe damage to the sensory nervous system, 254-UVC leads to more profound chromosomal condensation defects and reduced egg survival [82]. UV light at 254 nm was employed to induce heritable chromosomal rearrangements in *C. elegans*. Following exposure to a dose of 120 J/m^2^, a recovery rate of 3% was observed for these rearrangements. Interestingly, a substantial fraction of the induced mutations was not limited to simple intragenic lesions, such as point mutations, but included more complex structural changes such as deficiencies, which are chromosomal deletions resulting in the loss of several adjacent genes. Other rearrangements observed included translocations and duplications [83]. Moreover, Li et al. identified 57 intermediate-sized non-coding RNAs (is-ncRNAs) associated with the UV-induced DNA damage response. Among these, several is-ncRNAs showed a significant upregulation following UV irradiation, indicating their active role in the organism’s response to UV stress. These findings suggest that non-coding RNAs play a crucial, but previously underestimated, role in modulating the DNA damage response and contributing to UV resistance. The upregulated is-ncRNAs may have various functions, such as regulating gene expression, stabilizing damaged DNA, or interacting with repair proteins, adding another level of regulation to the DNA repair process. Further research into these is-ncRNAs could reveal novel targets for enhancing UV resistance in other organisms, including humans [79]. The identification of a *C. elegans* homolog of the UVSSA gene, associated with UV hypersensitivity syndrome in humans, provides new insights into the genetic basis of light intolerance. *C. elegans* lacking three DNA damage response pathways exhibited increased light intolerance, underscoring the link between effective DNA repair and resistance to light-induced stress. This finding suggests that UVSSA and its associated pathways play a critical role in protecting against the harmful effects of UV radiation. The study also underscores the importance of understanding how multiple DNA repair pathways interact to maintain cellular homeostasis under UV stress. The increased light intolerance observed in DNA repair-deficient mutants further emphasizes the need for a fully functional DNA damage response to cope with environmental light exposure, which may have implications for understanding similar conditions in humans [84]. Overall, the interplay of various DNA repair pathways, chromatin remodeling proteins, and regulatory RNAs underscores the complexity of the UV-induced DNA damage response in *C. elegans*, ensuring genome stability and cell survival under genotoxic stress and studies using *C. elegans* as a model in photoaging are summarized in Table 2.

## 4. Summary and Perspectives

*Caenorhabditis elegans*, a model organism with a long-standing reputation in the scientific community, has been of immense importance in elucidating the complex molecular processes underlying aging. This comprehensive review examines two key aspects of the aging process in *C. elegans*: chronological aging, characterized by a gradual deterioration of physical functions, and photoaging, triggered by exposure to UV radiation. These processes are regulated by a complex interplay of genetic factors and environmental influences. The study of chronological aging in *C. elegans* has elucidated fundamental mechanisms that not only improve our understanding of aging in this model organism but also offer promising insights into aging in more complex species. These findings may help to develop strategies that promote healthy longevity and mitigate the negative effects of aging and photoaging. However, it is essential to recognize the limitations of using *C. elegans* in aging research. While *C. elegans* has a relatively simple biology that enables rapid and cost-effective experiments, it lacks many physiological and anatomical features of higher organisms, such as complex organs and tissues. These distinctions limit the direct application of findings to aging in mammals, which is often gradual and multifactorial. Additionally, environmental influences on aging, such as UV-induced photoaging, may not be fully representative of human aging due to differences in skin structure and UV protection mechanisms. These factors emphasize the need for complementary studies in more complex model systems to validate and extend the insights gained from *C. elegans* research. Despite its limitations, the contribution of *C. elegans* to our understanding of the aging and photoaging process is still beyond question.

## Figures and Tables

**Table 1:** Key genes involved in aging in *C. elegans*: human orthologs, and functional insights.

*C. elegans* Gene	Human Ortholog	Function	Reference
*daf-2*	Insulin receptor/IGF-1 receptor	Receptor in the insulin-like signaling (IIS) pathway, negatively regulates lifespan	[27]
*age-1*	PI3K	Downstream of *daf-2*, negatively regulates lifespan	[27]
*daf-16*	FOXO	Transcription factor, positively regulates lifespan; master regulator of IIS	[35,36]
*utx-1*	UTX	Regulating lifespan through DAF-16-mediated pathway	[19]
*lys-7*	Lysozyme	Encodes lysozyme, important for antimicrobial activity and immunity	[37]
*skn-1*	Nrf2	Stress-responsive gene	[33,38]
*pmk-1*	p38 MAPKs	The p38 MAPK pathway	[39]
*jnk-1*	JNK	Homolog of JNK, the c-Jun N-terminal kinase (JNK) of the MAP kinase superfamily	[40]
*jkk-1*	JNK kinase	JKK-1 is a member of the MAP kinase kinase superfamily	[40]
*sirt-2.1*	SIRT-1	Regulates stress response, energy level, and longevity	[41]
*hcf-1*	HCF	host cell factor; a nuclear co-repressor of DAF-16	[17,42]
*akt-1*	AKT serine/threonine kinase 1	Regulates longevity, growth, metabolism	[43]
*akt-2*	AKT serine/threonine kinase 2	Regulates longevity, growth, metabolism	[43]
*let-363*	mTOR	Regulates longevity, growth, metabolism	[44]

**Table 2:** Summary of UV-induced photoaging studies of impaired DNA repair and molecular mechanisms in *Caenorhabditis elegans*.

UV Type	Main Biological Responses	Key Findings	Differences	References
UV	DNA damage, mutation rates, lifespan changes, antioxidant response	- UV-induced mutations (recessive lethal, chromosomal rearrangements); - UV radiation activates the PMK-1/p38 MAPK pathway trough JKK-1 and MOM-4. - Age mutants (*daf-16*) resist UV stress, extended lifespan; - UV sensitivity reduced in *rad-3* mutants; - A homolog of UVSSA gene associated with UV-hypersensitivity syndrome was identified; - Didymin protects *C. elegans* from UV stress by reducing ROS.	- Varied sensitivity in mutants. UV impacts lifespan and mutation rate primarily via DNA repair mechanisms; - UV radiation activates the PMK-1/p38 MAPK pathway via JKK-1 and MOM-4.	[9,56,74,77,79,83,84,85,86,87,88,89,90,91,92,93,94,95,96,97,98]
UVA	Collagen damage, behavior changes, apoptosis	- UVA accelerates aging via insulin-like signaling pathway. Antioxidants (green tea, naringin) protect against UVA damage; - Upregulation of apoptosis genes (*ced-3*, *ced-4*); - DAF-16, a critical transcription factor, plays a key role in stress resistance and longevity under UVA exposure.	Collagen and neural damage and apoptosis genes were explored.	[8,75,76,78,99]
UVB	Antioxidant response, lifespan extension	- *Cornus officinalis* extract (COE) protects from UVB damage via SKN-1/Nrf2 pathway; - Increased antioxidant activity and reduced ROS levels.	Activates antioxidant pathways, increasing lifespan via SKN-1/Nrf2 pathway.	[73]
UVC	Germline apoptosis, DNA damage, nervous system damage	- UVC induces germline apoptosis via NER pathway; - Far-UVC causes severe neuron damage; - Oxidative stress leads to nematode deactivation.	Non-study-specific oxidative stress pathway and antioxidant protective pathway.	[77,80,81,82,100]
Visible Light	Photooxidative stress, lifespan reduction	- Visible light reduces lifespan, causes photooxidative stress, and triggers unfolded protein response.	Non-UV-specific stress pathways (photooxidative stress, protein unfolding).	[101]
